# Surviving Separation: Laryngotracheal Injury in Blunt Neck Trauma—A Case Report and Review

**DOI:** 10.1155/crot/1481731

**Published:** 2026-08-20

**Authors:** Reggie John, Leonard Soh, Lilleen Huang, Duncan Wong, Xinyong Huang

**Affiliations:** ^1^ Department of Otorhinolaryngology, Khoo Teck Puat Hospital, Singapore, ktph.com.sg; ^2^ ASCENT Ear, Nose & Throat Specialist Group, Singapore

**Keywords:** laryngeal trauma, laryngotracheal injury, laryngotracheal separation, laryngotracheal trauma

## Abstract

Laryngotracheal trauma is a rare but fatal cause of injury in patients involved in polytrauma. Most patients succumb on site due to airway compromise. Thus, a delay in instituting interventions in such patients often leads to poor outcomes. In the handful that do make it to the hospital, there is a paucity of literature with regard to the optimal management of such conditions. In this case report, we present our management approach and highlight the dangers of dealing with such a case. We present a case of a young Asian male with complete laryngotracheal transection following blunt trauma in a high‐speed road traffic accident. Early and accurate diagnosis with expedient management by a multidisciplinary and experienced trauma team is paramount. We also discuss the salient points and pitfalls from initial assessment and stabilization of the patient to the technical details on the definitive management, as well as review the current literature in dealing with such an injury.

## 1. Introduction

Laryngotracheal trauma is a rare but fatal cause of injury in polytrauma patients. Complete laryngotracheal separation (CLS) is even more rare with a reported incidence of 1 in 4491 trauma admissions [1]. It is the second most common cause of death in patients with head and neck trauma after those with intracranial injuries [2]. In particular, “clothesline” injuries have been associated as the main cause of CLS [3]. Most patients succumb on site due to airway compromise. Thus, a delay in instituting interventions in such patients often leads to poor outcomes. Laryngotracheal trauma remains rare because of the protected position of the larynx. The larynx is shielded inferiorly by the sternum, superiorly by the mandible, posteriorly by the cervical spine, and laterally by the sternocleidomastoid muscles [2]. Due to the rarity of laryngotracheal trauma and the disproportionate appearance of the patient’s injuries [4], these conditions are frequently missed during the initial evaluation.

## 2. Case Report

**FIGURE 1:**
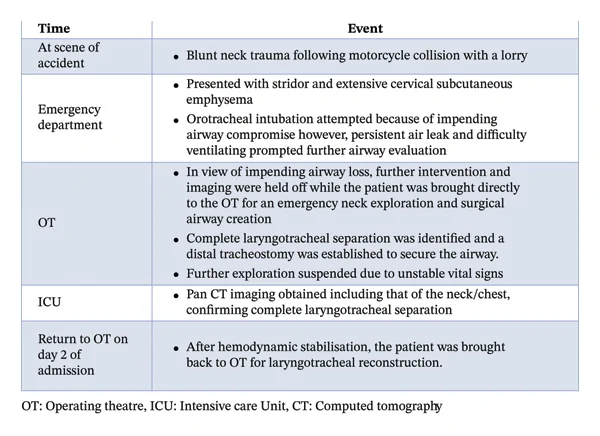
Airway and operative timeline.

A 21‐year‐old motorcyclist presented to the emergency department (ED) following a road traffic accident (RTA) with a lorry. He had a Glasgow Coma Scale (GCS) of E3V2M6. It was noted that he was stridorous with palpable subcutaneous emphysema over the neck. Oxygen saturations were 92% while on a nonrebreather mask with 100% oxygen. Oral endotracheal intubation was attempted by the ED physicians; however, persistent air leakage and difficulty ventilating was noted. A flexible scope was passed through the endotracheal tube (ETT) which revealed the distal end of the ETT abutting tissue, with the rest of the lower airway not visualized. He also had concurrent injuries of a pneumothorax and multiple limb fractures. He was subsequently listed for an urgent neck exploration and tracheostomy creation in the operating theater (OT). A figure illustrating the full airway and operative timeline is shown in Figure 1.

Intraoperatively, complete transection of the trachea was observed at the level of the cricoid through the Proximal 3 tracheal rings with the distal end of the ETT seen exiting the remnant proximal segment of laryngeal complex. The airway was secured with a tracheostomy tube inserted into the remnant distal trachea. Comminuted tracheal fragments were interspersed between the proximal and distal laryngotracheal segments with a gap of 4 cm possibly representing cartilage loss. Further exploration was suspended due to unstable vital signs.

He was then stabilized and transferred to the intensive care unit (ICU). Subsequent CT imaging of the neck showed extensive subcutaneous emphysema in the neck and face outlining the deep neck spaces. The airway below the level of the nasopharynx was effaced by a mixture of secretions and extensive subcutaneous emphysema. The laryngeal cartilages were severely disrupted with only part of the thyroid and cricoid cartilages visualized, with a fracture through the posterior midline of the cricoid.

After the patient was stabilized, he was brought back to the OT for definitive management at 48 h postadmission with a formal examination under anesthesia, laryngotracheal anastomosis, and a direct laryngoscopy.

**FIGURE 2:**
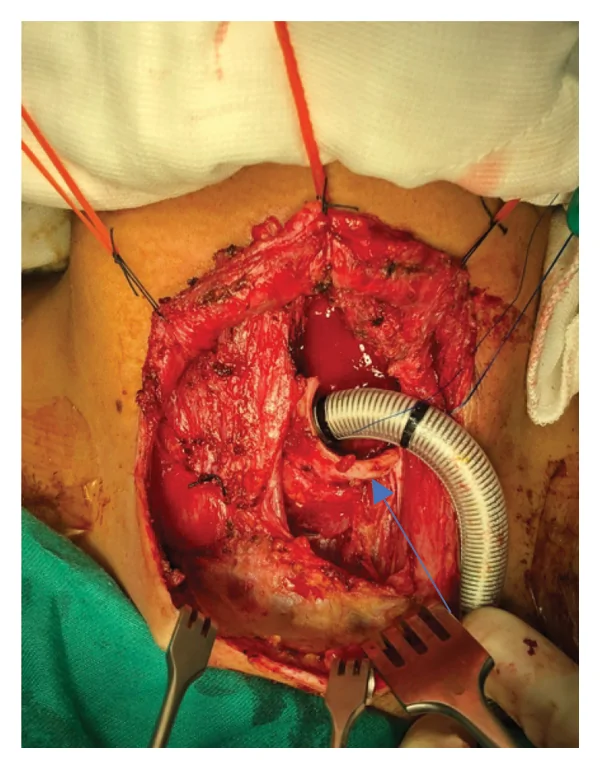
Tracheostomy tube removed and replaced with an endotracheal tube inserted into the distal trachea. Blue arrow: Fractured anterior half of cricoid cartilage attached to the distal tracheal segment.

**FIGURE 3:**
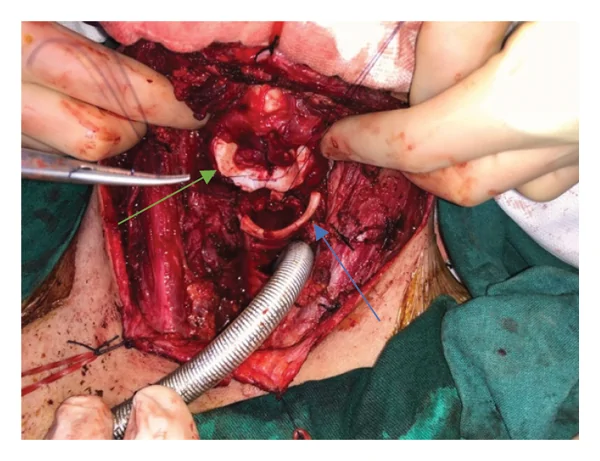
Fractured cricoid cartilage with the separation of distal tracheal and proximal laryngeal segments. New tracheostomy window created between the 4th and 5th tracheal rings. Blue arrow: Fractured anterior half of cricoid cartilage. Green arrow: Posterior half of cricoid cartilage.

**FIGURE 4:**
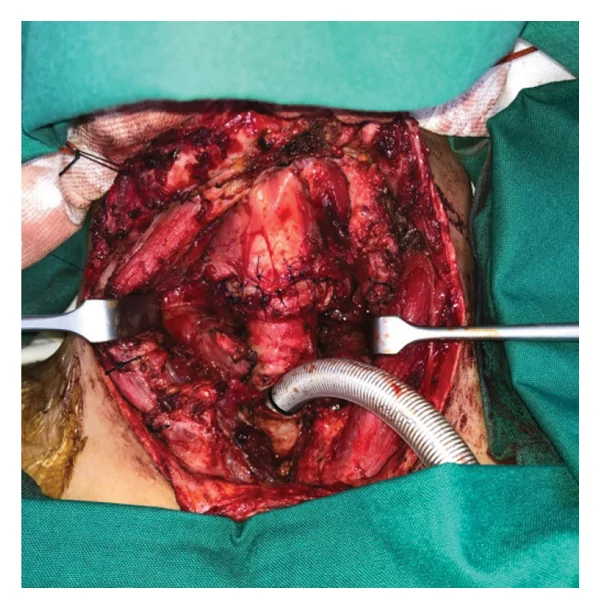
Laryngotracheal complex postreconstruction with PDS 4/0 sutures.

Intraoperatively, both vocal cords appeared intact with no mucosal injury or hematoma. CLS was seen between the thyroid and cricoid cartilages with the dominant posterior half of the cricoid still attached to thyroid cartilage and arytenoids and the anterior half attached to the distal trachea, as shown in Figures 2 and 3. A new tracheostomy window was created between the 4th and 5th tracheal rings. The recurrent laryngeal nerves were not identified. Then, the posterior tracheal wall was sutured to the distal end of the posterior half of cricoid cartilage with PDS 4/0. Two‐halves of cricoid cartilage were reanastomosed at their lateral ends with interrupted PDS 4/0. The superior edge of the anterior half of the cricoid cartilage was sutured to the inferior edge of the thyroid cartilage with PDS 4/0. Mucosa‐to‐mucosa approximation was successfully achieved, as shown in Figure 4, with best attempts made to approximate the remnant perichondrium and surrounding muscular soft tissue over areas of cartilage tissue loss. As a tension‐free anastomosis was achieved, a decision was made not to use an intraluminal stent, thereby also avoiding any stent‐related morbidity. A drain was inserted into the tracheal gutter. Grillo stitches were inserted to discourage extension of the neck and maintain it in a flexed position.

**FIGURE 5:**
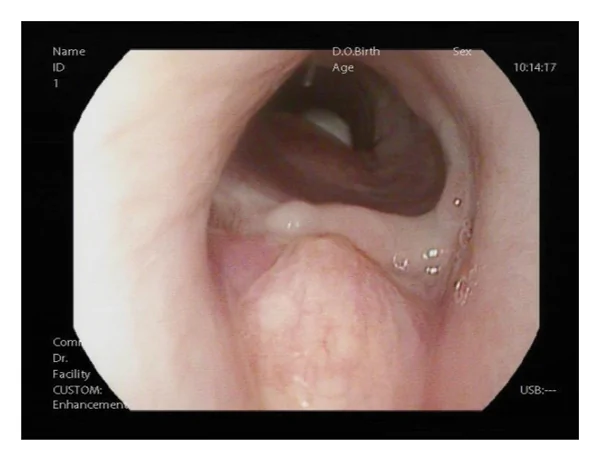
Tracheoscopy at 6 weeks postoperatively showing an intact laryngotracheal complex with granulation tissue at the anastomotic site.

His postoperative recovery was uncomplicated. Tracheoscopy done 6 weeks postoperatively showed an oval‐shaped subglottic airway (Cotton–Myer Grade 1) with minimal granulation noted along the anastomosis, as shown in Figure 5. In addition, the right vocal cord was mobile though the left was not. Eventually, he was decannulated 10 weeks postoperatively. Voice outcomes were fair at G1B1, the left vocal cord remained immobile at a level mismatch with the contralateral side but with satisfactory compensation of right vocal cord movements at 6 months postinjury.

## 3. Discussion

Owing to the uncommon presentation of CLS, such cases present a diagnostic challenge during the initial assessment. The mechanism of injury (MOI) can be broadly divided into external trauma or internal injuries. External trauma can include blunt trauma such as hanging/strangulation, clothesline injury, or a high‐speed RTA, as well as penetrating injuries such as knife attacks or bullet wounds. Internal injuries can be iatrogenic (such as repeated attempts at intubation), foreign body ingestion, thermal injuries, or caustic ingestion. The MOI has an impact on the extent and severity of the laryngeal injury sustained. In the case of an RTA, it is crucial to know the speed and configuration of the motor vehicles involved as well as the use of helmets or seatbelts. It has been noted that clothesline injuries specifically may be an important cause of major neck trauma [5].

Common findings noted in initial assessment may include stridor, voice hoarseness, hemoptysis, neck swelling, and dyspnea. In our case, the patient was stridorous with palpable subcutaneous edema at the onset, with mild bruising over the neck. After attempts at blind intubation in the ED, it was noted that the subcutaneous edema over the neck and thorax worsened, and there was persistent air leakage. In cases of CLS, spontaneous ventilation may initially preserve distal airflow through partial soft tissue continuity and negative intrathoracic pressure gradients. However, once converted to positive pressure ventilation following sedation or intubation, positive pressure may preferentially direct air into disrupted soft tissue planes rather than the distal airway, exacerbating subcutaneous emphysema and pneumomediastinum. Additionally, multiple intubation attempts could potentially exacerbate an unstable laryngeal fracture into a full CLS. These factors likely contributed to the observed worsening findings in our patient.

A literature search was performed using the PubMed (MEDLINE) database to identify published case reports and case series of CLS following blunt neck trauma. Search terms included combinations of *laryngotracheal separation*, *CLS*, *laryngotracheal transection*, *laryngotracheal disruption*, *cricotracheal separation*, *laryngotracheal injury*, *laryngotracheal trauma*, and *laryngeal trauma*. The search was limited to English‐language case reports and case series involving adult human subjects published in the last 15 years between 2011 and 2026. Reference lists of relevant articles were also manually screened to identify additional eligible publications. Reports lacking sufficient detail regarding reconstructive management or postoperative outcomes were excluded.

In other studies, physical examination findings can vary. In addition to the abovementioned symptoms, others include dysphonia, ecchymosis, or loss of laryngeal landmarks [2]. Kubota et al. [4] reported an abnormal see‐saw movement of the anterior neck. Elbadan et al. [3] reported in a retrospective study of 23 patients with cricotracheal separation that 78.26% of patients presented with subcutaneous emphysema within the first 24 h, suggesting that the presence of subcutaneous emphysema should give a high suspicion of laryngeal injury. In our selected cases, as seen in Table 1, subcutaneous emphysema occurred in almost all patients with CLS.

**TABLE 1:** Review of selected complete laryngotracheal separation case reports in the literature.

Author & year	Country	Mechanism of injury	Symptoms & findings	Surgical findings	Intervention performed	Outcomes
Hashima et al. 2011 [6]	Malaysia	RTA	RD, neck swelling and SE	CLS	Tracheostomy, thyrotracheal anastomosis with 3/0 nonabsorbable sutures	Survived, decannulated with a weak voice (bilateral VC immobility but with an adequate airway)

Singh et al. 2012 [7]	India	Self‐inflicted knife injury	Open neck wound	CLS, esophageal anterior wall perforation	Tracheostomy, primary repair of esophagus and primary end‐to‐end anastomosis of the tracheal injury	Survived, still on tracheostomy

Malliari et al. 2014 [8]	Greece	RTA	Neck abrasions and SE	CLS, comminuted cricoid cartilage fracture	Laryngotracheoplasty, Montgomery T‐tube used as a stent	Survived, no stenosis, unilateral VC palsy

Vivero et al. 2014 [9]	USA	Gunshot Wound	Open neck wound	CLS, thyroid cartilage fracture, laryngeal fractures	Laryngeal fractures reduced and fixated with Prolene 2/0 sutures, Tracheostomy with a Bjork flap performed, Cricotracheal reconstruction with Vicryl 3/0, stent placed in subglottis and upper trachea, local muscle flap placed over exposed airway	Survived, decannulated, recovery from unilateral VC paralysis to paresis

Ahn et al. 2015 [10]	Korea	Attempted suicide by stabbing	Not mentioned	CLS, incomplete separation of thyroid and cricoid cartilages, bilateral RLN transection, loss of tracheal rings	Tracheostomy, thyrocricoid separation was reconstructed with Prolene 3/0 and cricotracheal anastomosis between the cricoid cartilage and the fifth tracheal ring was performed using Vicryl 4/0 sutures Transverse laser cordotomy performed at 5 weeks postinjury	Survived, decannulated at 7 weeks postinjury

Ershadi et al. 2017 [11]	Iran	RTA with strangulation	Skin contusion, SE	CLS	Primary cricotracheal anastomosis performed	Survived

Plocienniczak et al. 2019 [12]	USA	Knife wound	Multiple open neck wounds	CLS, near complete esophageal transection	Tracheostomy, primary repair of esophagus, and cricotracheal anastomosis with Prolene 2/0 sutures	Survived, Decannulated 8 months after injury, VCs remain immobile, developed subglottic stenosis requiring 2 CO2 laser dilatations at 2 and 6 months postinjury

Elbadan et al. 2020 [3]	Egypt	Various methods of blunt trauma to the neck, RTA, physical assault	RD, stridor, voice hoarseness	Partial and CLS, thyroid prolapse, cricoid fracture, pharyngeal tear	Tracheostomy, cricotracheal repair with Vicryl 4/0 and 3/0 sutures with suprahyoid release	All survived. Out of 30% of patients who needed tracheostomy, 85.75% of them were decannulated.

Rozhan 2021 [13]	Malaysia	RTA	RD, SE, dysphonia, desaturation	CLS, left cricoid fracture, thyroid gland severed	Tracheostomy, cricoid fracture repositioned and sutured with Dafilon 2/0. Anastomosis of cricoid and trachea done with Dafilon 2/0	Survived, still on tracheostomy

Ciburaite and Kaseta 2022 [14]	Lithuania	RTA	Aphonia, SE	CLS	Tracheostomy, delayed tracheal resection and anastomosis after 2 weeks	Survived, decannulated following 4 months with fixed VC bilaterally

Ebrahimian et al. 2023 [15]	Iran	RTA	RD, SE, desaturation	CLS, esophageal transection	Tracheostomy, primary repair of esophagus and cricotracheal anastomosis with absorbable 3/0 sutures. SCM muscle flap placed between the esophagus and trachea	Survived, decannulated at 4 weeks, VCs mobile bilaterally

Soni et al. 2025 [16]	India	Accidental strangulation	Multiple neck abrasions, RD, SE	CLS, esophageal transection	Tracheostomy, primary repair of esophagus, and cricotracheal anastomosis with PDS 3/0.	Survived, developed Grade 2 subglottic stenosis with fixed VC bilaterally

Uniyal et al. 2025 [17]	India	RTA	RD, SE, desaturation	CLS	Tracheostomy, cricotracheal anastomosis with Prolene 2/0 sutures	Survived

Abbreviations: CLS, complete laryngotracheal separation; RD, respiratory distress; RLN, recurrent laryngeal nerve; RTA, road traffic accident; SE, subcutaneous emphysema; VC, vocal cord.

When the patient is in the ED, it is crucial to perform primary and secondary surveys so as not to miss concomitant injuries. When securing the airway, oral intubation should be approached with caution in a patient with suspected laryngeal trauma. Schaefer et al. [18] formulated three criteria to decide whether to proceed with oral intubation in a patient with blunt neck trauma. These were that the larynx and trachea must be clearly intact and in continuity, the airway should be visible to direct inspection by endoscopy, and finally that the intubation should be performed by a highly experienced airway physician. Management in such scenarios should involve close collaboration and communication between experienced airway physicians and surgical teams. Ideally, in cases of suspected airway instability, intubation attempts should be performed by a highly experienced anesthetist. Unsuccessful repeated attempts at intubation could potentially worsen laryngotracheal injury and destabilize the airway with catastrophic consequences [18]. Nasoendoscopic visualization of the airway can be used to help the managing team identify that they are dealing with a laryngeal injury should there be mucosal edema, lacerations, or vocal cord immobility. Otherwise, in an impending airway situation, there should be a low threshold to proceed with an awake “slash” tracheostomy. It is also the opinion of the authors that a cricothyroidotomy in such instances may be complicated and risk losing the airway due to the limited exposure and immediate entry into a fractured surgical field, which would make identification of structures challenging thereby potentially missing a distal cricotracheal separation.

Imaging should ideally be performed with a CT of the neck and thorax as it will be sufficient to diagnose most laryngeal fractures and dislocations [19] and aid in surgical planning. It may also be useful to investigate for occult injuries that may not have been picked up clinically. In addition, laryngeal trauma can be associated with concomitant cervical spine injuries in 10% of cases [20].

Then, the patient should be brought to the OT within 24–48 h of the initial injury [6, 21]. This is to reduce the risk of infection, subglottic stenosis, granulation and scar tissue formation, and airway edema.In our cases reviewed, all underwent neck exploration and primary repair within this period with the exception of Hashima et al., who reported only having identified a laryngotracheal separation 25 days postinjury [6], and Ciburaite and Kaseta, who delayed repair till 2 weeks postinjury [22]. For a full cricotracheal separation, a tracheostomy should be fashioned or moved to a lower tracheal insertion site prior to anastomosis. An attempt should be made to examine other anatomical structures, especially the pharynx and great vessels of the neck [3]. Repair should begin with a careful posterior anastomosis with interrupted sutures before proceeding to the anterior tracheal repair to avoid obscuring separation. The use of an intraluminal stent may be considered, particularly in patients with a crush injury. In addition, where due to extensive cartilage loss, there is difficulty in ensuring tension‐free anastomosis, release techniques, such as suprahyoid muscle tissue release [22], proximally as well as distal segment tracheal tissue release, can help approximate the separate segments and improve outcomes of the anastomosis.

In the cases reviewed in Table 1, all patients survived with most who were tracheotomized eventually decannulated albeit with some long‐term morbidity. These included poor voice with permanent unilateral or bilateral vocal cord paresis, granulation tissue formation, and airway stenosis [23].

Finally, we suggest the following steps of assessment for patients who present with a history of blunt neck trauma and suggestive examination findings.

1. Approach blind oral endotracheal intubation with caution, particularly in a patient with suspected laryngeal injury. Flexible nasoendoscopic assessment can be helpful to cue the team in that they may be dealing with a laryngeal injury if there are no other obvious external physical signs. Alternatively, awake fiberoptic intubation can be considered in selected cases and should be performed by skilled airway physicians so as not to exacerbate airway injuries.
2. The choice of surgical airway is an urgent tracheostomy creation to minimize aggravation of the airway, preferably in the controlled setting of the OT.
3. Imaging is encouraged as this provides a better assessment of the extent of injury and aid in definitive surgical management.
4. Neck exploration should be performed within 24–48 h after presentation to assess for concomitant injuries in the neck and to perform the appropriate repair as necessary.

## 4. Conclusion

This case reinforces the need for a high degree of suspicion in patients with blunt neck trauma to rule out potential laryngeal injury. Airway management in CLS is uniquely challenging and carries significant risk of precipitating rapid deterioration, in which the risks of intubation must be weighed against a more preferable alternative of surgical airway creation. Finally, early intervention and laryngeal reconstruction should be undertaken expediently so as to improve overall outcomes.

## Funding

The authors have nothing to report.

## Consent

Written informed consent was obtained from the patient for publication of case details and accompanying images.

## Conflicts of Interest

The authors declare no conflicts of interest.

## Data Availability

Data sharing was not applicable to this article as no datasets were generated or analyzed during the current study.
